# *Dirofilaria repens* Nematode Infection with Microfilaremia in Traveler Returning to Belgium from Senegal

**DOI:** 10.3201/eid2409.180462

**Published:** 2018-09

**Authors:** Idzi Potters, Gaëlle Vanfraechem, Emmanuel Bottieau

**Affiliations:** Institute of Tropical Medicine Antwerp, Antwerp, Belgium (I. Potters, E. Bottieau);; Centre Hospitalier Interrégional Edith Cavell Hospital Group, Brussels, Belgium (G. Vanfraechem)

**Keywords:** Dirofilaria repens, nematode, parasites, eye worm, human infection, ocular infection, filariasis, conjunctiva, microfilaremia, microfilariae, blood, traveler, zoonoses, Senegal, Belgium

## Abstract

We report human infection with a *Dirofilaria repens* nematode likely acquired in Senegal. An adult worm was extracted from the right conjunctiva of the case-patient, and blood microfilariae were detected, which led to an initial misdiagnosis of loiasis. We also observed the complete life cycle of a *D. repens* nematode in this patient.

On October 14, 2016, a 76-year-old man from Belgium was referred to the travel clinic at the Institute of Tropical Medicine (Antwerp, Belgium) because of suspected loiasis after a worm had been extracted from his right conjunctiva in another hospital. Apart from stable, treated arterial hypertension and non–insulin-dependent diabetes, he had no remarkable medical history. For the past 10 years, the patient spent several months per year in a small beach house in Casamance, Senegal, and did not travel to any other destination outside Belgium. His last stay in Senegal was during October 2015–May 2016, during which time he took care of dogs roaming on the beach.

On September 30, 2016, unilateral right conjunctivitis developed in the patient, and he was referred to an ophthalmologist, who extracted a worm (length 10 cm, diameter 470 μm) (Figure, panel A). The patient did not report any previous symptoms such as itching, larva migrans, or migratory swelling.

**Figure:**
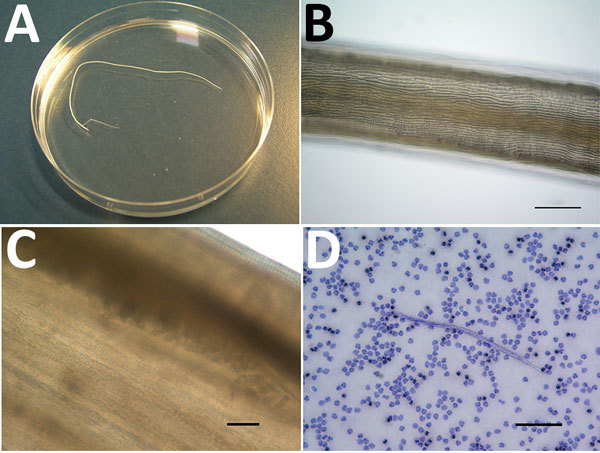
*Dirofilaria repens* adult worm isolated from the right conjunctiva of a 76-year-old man who returned to Belgium from Senegal, and microfilaria detected by using the Knott test. A) Macroscopic image of the adult. B) Microscopic image of the adult cuticle, showing the typical longitudinal ridges. Scale bar indicates 200 μm. C) Eggs in utero, indicating that the adult is a gravid female worm. Scale bar indicates 50 μm. Panel C has been cropped and contrast was increased to improve visibility of eggs. D) Microfilaria found in the blood of the patient. In a Knott test, microfilariae usually appear stretched out and slightly longer than those observed in a Giemsa-stained blood film. Scale bar indicates 100 μm.

Results of a physical examination were unremarkable. Blood analysis showed a leukocyte count of 8,330 cells/μL and 16.8% eosinophils. All other first-line laboratory parameters, including total level of IgE, were within reference ranges. A pan filaria IgG-detecting assay (*Acanthocheilonema viteae* ELISA Kit; Bordier Affinity Products SA, Crissier, Switzerland) showed a positive result. All other relevant serologic assays showed negative results. Blood smear examination after Knot concentration showed 6 microfilariae of *Dirofilaria* sp./mL of blood. 

Although treatment for such infections is not well established, the patient was given ivermectin (200 μg/kg, single dose) on October 15. The patient had general itching and fever (temperature up to 40°C) the next day. Blood test results on October 26 showed a leukocyte count of 8,410 cells/μL and 27.9% eosinophils. The patient recovered uneventfully. In September 2017, the patient was free of symptoms, and his eosinophil count was 470 cells/μL.

Human dirofilariasis is a mosquitoborne zoonosis caused by filarial worms of the genus *Dirofilaria*, which has 2 subgenera: *Dirofilaria* (the most common species is *D. immitis*) and *Nochtiella* (the most common species is *D. repens*). The main clinical manifestations are subcutaneous or ocular nodules, and a diagnosis is usually made by biopsy or worm extraction. The risk for humans to acquire dirofilariasis has increased because of climate changes and larger distribution ranges of vectors (*1*).

Human dirofilariasis is currently considered an emerging zoonosis (*2*). *D. repens* nematodes have a large geographic distribution that includes Africa, Asia, and Europe and have recently spread into colder regions (*3*). Studies of primates indicate that *D. repens* nematodes need to develop for ≈25–34 weeks before they are fully mature and produce microfilariae (*4*). This finding suggests that the patient we report acquired the infection in Senegal, possibly through close contact with dogs.

Initially, loiasis was suspected as a diagnosis, given the location of the adult worm and presence of microfilaremia. However, the length (10 cm) of the adult worm did not correspond to a *Loa loa* worm, which can reach a maximum length of ≈7 cm. Microscopic examination of the cuticle identified conspicuous longitudinal ridges, which are typical for certain *Dirofilaria* spp. but absent in *L. loa* worms (Figure, panel B). These ridges also ruled out *D. immitis* worms.

When we took the largest diameter of the adult worm (470 μm) into account, we made a diagnosis of *D. repens* nematode infection (*5*). Eggs found in utero (Figure, panel C) confirmed that the worm was a gravid adult female. This diagnosis was supported by morphologic features of the blood microfilariae: terminal extremities that did not contain nuclei (*L. loa* microfilariae have nuclei extending to the tip of the tail) and short cephalic spaces containing 2–4 nuclei (Figure, panel D; Technical Appendix Figure). We measured 25 larvae, and they had an average length of 376 μm (range 357–395 μm) and an average diameter of 9.7 μm (range 7.5–10.0 μm), all features compatible with *D. repens* microfilariae (*6**,**7*).

We attempted to provide molecular confirmation of the infecting species by using 2 PCRs: 1 reported by Gioia et al. in 2010 (*8*) and 1 reported by Latrofa et al. in 2012 (*9*). Both techniques, which were performed with material from the adult worm, did not confirm identification of infecting species, probably because of prolonged preservation of the worm in formaldehyde.

*D. repens* worms seldom fully develop and produce microfilariae in humans. To our knowledge, 5 such cases have been reported: 3 with microfilariae in tissues surrounding adult worms and 2 with microfilariae in blood (*10*). There might have been immune impairment in our patient with diabetes, which enabled completion of the worm cycle, a phenomenon also observed in macaques with decreased immunity (*4*).

In conclusion, this case highlights the need for careful parasitologic examination when clinical and laboratory findings (i.e., presence of an eye worm and microfilaremia) lead to a diagnosis that is epidemiologically unexpected. In addition, clinicians should be aware that similar clinical presentations might also be increasingly observed in nontropical settings.

Technical Appendix Additional information on *Dirofilaria repens* infection with eye worm and microfilaremia in traveler returning from Senegal to Belgium.
