# Supplementary material for: Dirofilaria repens Nematode Infection with Microfilaremia in Traveler Returning to Belgium from Senegal
Source: Emerg Infect Dis. 2018 Sep;24(9):1761–3. doi: 10.3201/eid2409.180462 (PMC6106435; doi:10.3201/eid2409.180462)
Supplement: Technical Appendix — Additional information on Dirofilaria repens infection with eye worm and microfilaremia in traveler returning from Senegal to Belgium. [file 18-0462-Techapp-s1.pdf]

Article DOI: http://doi.org/eid2409.180462

Dirofilaria repens Nematode Infection with Microfilaremia in Traveler Returning to Belgium from Senegal

Technical Appendix.

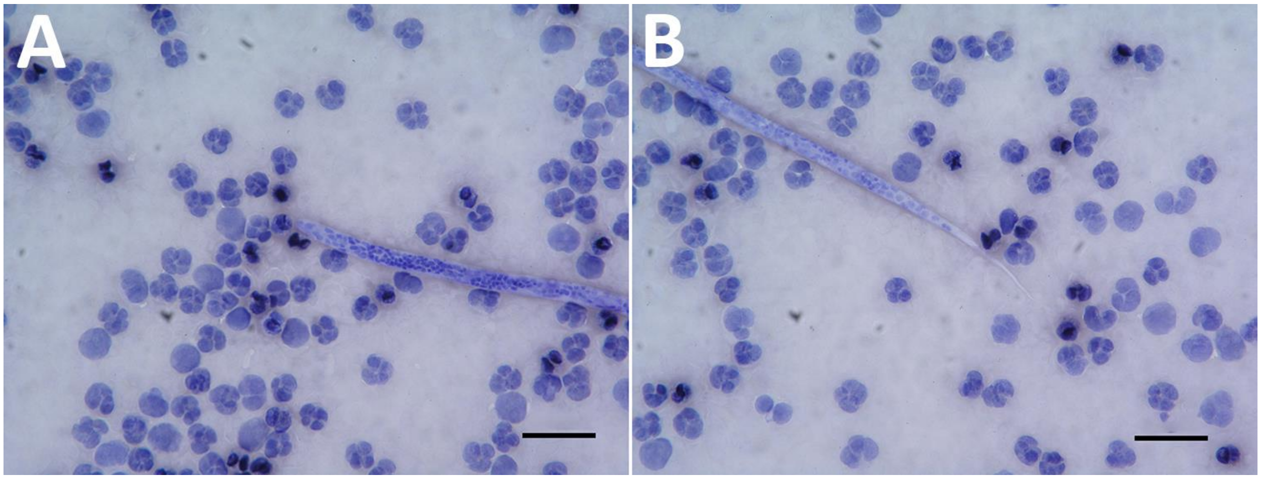

Technical Appendix Figure. *Dirofilaria repens* microfilaria in blood of a 76-year-old man who returned to Belgium from Senegal. A) Cephalic end of microfilaria. B) Tail end of microfilaria. Scale bars indicate 30 µm, original magnification ×500.
